# Ancient DNA study resolves the taxonomic status of the extinct large Palaearctic crane *Grus primigenia* Milne-Edwards, 1871 (Aves: Gruiformes: Gruidae)

**DOI:** 10.1098/rsos.260699

**Published:** 2026-10-28

**Authors:** John R. Stewart, Rebecca Rickard, Sheila Hamilton-Dyer, Keith Dobney, Veronique Laroulandie, Josep Antoni Alcover, Joan Femenias-Gual, Nadja Pöllath, Erik Ersmark, Bodil Cronholm, Per G. P. Ericson, Love Dalén

**Affiliations:** ^1^ Faculty of Health, Environment and Medical Sciences, Bournemouth University, Poole, UK; ^2^ Department of Archaeology, University of Aberdeen, Aberdeen, UK; ^3^ Department of Archaeology, Classics and Egyptology, University of Liverpool, Liverpool, UK; ^4^ Université de Bordeaux, CNRS, Ministère de la Culture, PACEA, UMR 5199, Pessac, France; ^5^ Department of Animal and Microbial Biodiversity, Mediterranean Institute of Advanced Studies (UIB-CSIC), María de Maeztu Center of Excellence, Mallorca, Spain; ^6^ Institut Català de Paleontologia Miquel Crusafont (ICP-CERCA), Universitat Autònoma de Barcelona, Barcelona, Spain; ^7^ Grup de Recerca de Ciències de la Terra, Departament de Biologia, Universitat de les Illes Balears, Palma, Spain; ^8^ Staatliche Naturwissenschaftliche Sammlungen Bayerns (SNSB)-Staatssammlung für Paläoanatomie München (SPM), Ponig, Germany; ^9^ Department of Bioinformatics and Genetics, Swedish Museum of Natural History, Stockholm, Sweden; ^10^ Centre for Palaeogenetics, Stockholm, Sweden; ^11^ Department of Zoology, Stockholm University, Stockholm, Sweden

**Keywords:** extinction, crane, size change, aDNA, Quaternary

## Abstract

The identity of the extinct large crane *Grus primigenia* has been debated for over 150 years. Alternative interpretations suggest that such large crane remains represent extant cranes such as the sarus crane *Antigone antigone*, the Siberian white crane *Leucogeranus leucogeranus* or simply larger individuals of common crane *Grus grus*. Morphometrics has shown that the common crane is sexually dimorphic today with larger males and smaller females. We analysed cranes from the Late Pleistocene to Late Holocene and found that the lack of smaller female-sized craneremains suggests that *G. grus* may have reduced in size over time. In the present study, we successfully performed an ancient DNA analysis to assess the phylogenetic identity of 18 crane remains from the Late Pleistocene of Spain and France and the Late Holocene of Britain and Germany. All remains were identified as *G. grus*. These ancient cranes included individuals that were larger than modern *G. grus* and ones that conformed in size with common crane today. Ancient DNA-based sexing found that all the bones that were larger than those of modern males were male, while specimens the size of modern males included ancient females. This confirmed that the ancient cranes were sexually dimorphic *G. grus* that have become smaller since the Saxon times (884 yr BP).

## 1. Introduction

The end of the Pleistocene (11.7 kyr BP) saw the extinction of many large mammal species (megafauna) across the world, and a diverse literature exists on their taxonomic affinities as well as the cause of these extinctions [1–4]. More recently, the precise timings of the extinctions of some of these taxa have been extended into the Holocene Epoch [5,6]. However, the comparative lack of studies on the extinction of other Late Quaternary vertebrate classes, such as birds [7], hampers a fuller understanding of the likely processes involved in these extinctions. In addition to mammals, several extinct bird taxa have been described from the Palaearctic although many of these may not be reliable [8]. The characters used to describe these extinct birds often involve size and morphometric shape differences when compared to their living congeners which is problematic as such characters are used to describe avian subspecies as well as species [8]. This means that there is no clear way of telling what taxonomic distinction a difference in size and shape represents.

The continental bird species with perhaps the longest-standing controversy in terms of its identity is the extinct large Palaearctic crane *Grus primigenia*. Next to the great auk, it is the most discussed extinct Quaternary bird species in the Palaearctic [9]. The species was originally named based on a fragment of tibiotarsus from Late Pleistocene cave deposits in France [10]. The distal tibiotarsus from the Grotte des Eyzies in Dordogne was described as wider mediolaterally than that of a sarus crane (*Antigone antigone*) [10]. Two further specimens, a humerus and a mandible, from the Grotte de la Madeleine and the Grotte Gourdan, respectively, were subsequently assigned to the species [11]. Since then, the taxon was either accepted [12–18], reinterpreted as belonging to the extant Asian sarus crane [19–27] or thought to be a larger version of the common crane (*Grus grus*) found in Europe today [28–35]. There is a slightly ambiguous viewpoint from Saint-Périer [13] in studying the La grotte de Gouërris crane tarsometatarsus which he described as *A. antigone* var. *primigenia* because it was slightly smaller than *A. antigone*. The possibility that the larger cranes in the British Isles may have belonged to a subspecies distinct from those in continental Europe has also been considered [31,33,35], particularly since the cranes from the Danish peats were not large [31,33], although large cranes had been found over much of northwestern Europe [31–33]. Harrison & Cowles [16] concluded that the large crane bones were unlikely to belong to *A. antigone* because the geographical distribution of this species is isolated and restricted to regions south of the Himalayas. Northcote & Mourer-Chauviré [17,18] undertook a detailed morphological comparison between *A. antigone* and *G. primigenia* in which they described characters that distinguished the two—although these were not applied to modern *G. grus* specimens. The possibility that some of these large crane remains, particularly those of Late Pleistocene age, belonged to the Siberian white crane (*Leucogeranus leucogeranus*) has also been considered [31,33,36].

In addition to *G. primigenia*, there have been two other extinct cranes named from Quaternary fossils in the Western Palaearctic, such as *Grus melitensis* Lydekker [37] from Malta and *Grus bohatshevi* Serebrovsilj [38] from Azerbaijan. The former was described as large and similar to *A. antigone* with morphological differences in the coracoid and tibiotarsus and was thought to be an island endemic restricted to Malta [19,37,39]. *Grus bohatshevi* meanwhile was described as similar to *L. leucogeranus* with differences in the skull [38].

The temporal and geographic range of *G. primigenia* is from the penultimate interglacial (MIS 7 at *ca* 246–186 kyr BP [40]) to the Late Holocene with find locations in France, England and Spain [33,36,41,42]. The oldest specimen attributed to *G. primigenia* is a distal left radius from the MIS 7 fluvial deposits of the Thames basin at Ilford [43] (figure 1; table 1), while the youngest are from the Glastonbury Lake Village in Somerset dated to the Iron Age (*ca* 2200–2000 yr BP [16]; table 1). However, the youngest site in Europe to have produced large cranes, regardless of their being identified as *G. primigenia*, is the Anglo-Saxon (1300–884 yr BP) site of Flixborough in North Lincolnshire, England (tables 1 and 2). These remains were not attributed to the extinct species instead described as likely large common crane possibly belonging to an extinct larger subspecies restricted to Britain [34,35].

**Table 1.** Sites with claimed *Grus primigenia*, *Antigone antigone* or crane remains thought to be larger than common cranes, including the material sampled here. ? = no record of skeletal element(s).

site	country	age	material	publication	notes
Les Eyzies, the Dordogne	France	Late Pleistocene	distal tibiotarsus	[10]	holotype of *G. primigenia*
Grotte de la Madeleine	France	Late Pleistocene	humerus	[11]	type of *G. primigenia*
Grotte Gourdan	France	Late Pleistocene	mandible	[11]	type of *G. primigenia*
La grotte de Gouërris (Haute-Garonne), Level B	France	Late Pleistocene	complete tarsometatarsus and a distal femur	[13]; identified by M. Boule	*Grus* cf*. primigenia* (Miln.-Edw.) [10] it was thought that 2 sized cranes had existed in the past (see main text)
La grotte des Harpons (Lespugne, Haute-Garonne), Level A	France	Late Pleistocene	distal extremity of tarsometatarsus^a^	[12]; identified by M. Boule	*G. primigenia*
**Le Morin, Gironde**	France	Late Pleistocene	proximal right femur	[15,44]	*G. primigenia*
**Grotte Tastet, Pyrénées-Atlantiques**	France	Late Pleistocene	right humerus shaft	[45]	large *G. grus*
Duruthy (Lande), Layer 3	France	Late Pleistocene C14–13.130 kyr BP [46]	2 proximal^a^ and 2 distal^a^ tibiotarsi	[15,47,48]	*G. primigenia*
Les Capins	France	?	distal humerus	[25,42]	*G. primigenia*
Cabezo Redondo, Villena, Alicante	Spain	Bronze Age	unspecified?	[28]	*G. primigenia*
**Es Pouàs, Sant Antoni de Portmany, Eivissa, Balearic Islands**	Spain	Late Pleistocene/Holocene	humerus, tibiotarsus, femur sampled in this study and many more	[49,50]	*G. primigenia*, later reidentified as large *G. grus*
Avenc De Na Corna (Mallorca, Balearic Islands)	Spain	Pleistocene	distal right humerus, distal right femur, distal right tibiotarsus, proximal phalange of the second pedal digit, two cervical vertebrae	[18,25,51]	*G. primigenial*
**Xanten, North Rhine-Westphalia**	Germany	Roman	femur, humerus	[52]	*G. grus*
**Pestenacker, Bavaria**	Germany	Neolithic	radius, phalanx manus	[53]	*G. grus*
Ehrenstein, Ulm	Germany	Neolithic	incomplete humerus and radius	[22]	*A. antigone* associated with a humerus of *G. grus*
Hohen Viecheln, Mecklenburg	Germany	Mesolithic	?	[23]	*A. antigone*
King's Cave on the shore of Loch Tarbert, Isle of Jura, Scotland	the UK	Late Bronze Age or early Iron Age	distal ulna	[16]	*A. antigone*
Ilford, Essex	the UK	MIS7	distal left radius^b^	[43]	*G. primigenia*
London Basin	the UK	Pleistocene	?	[16]	slightly larger than *G. grus*. May not be a separate site but refer instead to Ilford
Glastonbury Lake Dwelling, Somerset	the UK	Iron Age	right ulna	[16]	*G. primigenia* associated with *G. grus*
Meare Lake Village, Somerset	the UK	Iron Age	?	[54]	*G. grus* but possibly incorrectly reported as *G. primigenia* in [55]— supplementary material
**Cambridgeshire Fens**	the UK	Mesolithic to Bronze Age	various from associated skeletons	[26,33,56]	all *G. grus*
**Caerleon, South Wales**	the UK	Roman	various from associated skeletons	[29]	all large *G. grus*
Barking Abbey, London	the UK	Anglo-Saxon	unspecified	[57]	*G. primigenia*
Longthorpe, Peterborough, Cambridgeshire	the UK	Iron Age	unspecified	[58 in 57]	*A. antigone*
Austin Friars, Leicester	the UK	Medieval	unspecified	[59 in 57]	*Grus* sp. —larger than common crane
**Flixborough, Lincolnshire**	the UK	Anglo-Saxon	various from associated skeletons	[34,35]	all large *G. grus*

^a^ These bones show clear human modifications.

^b^ The distal left humerus reported from Ilford in [60] appears to be incorrect as no mention of this specimen is made elsewhere. Sites sampled in this study are in bold.

**Table 2.** The *Grus* samples analysed in this study with details of their skeletal element, location, age and where the specimens are housed.

laboratory number	sample number	site	age	skeletal element	museum collection
PE14	IMEDEA 106200	Es Pouàs, Eivissa, Balearic Islands, Spain (A5/B5, level 3, −180 cm)	Pleistocene to Holocene, probably Late Pleistocene	distal right humerus	Mediterranean Institute for Advanced Studies
PE15	IMEDEA 22338	Es Pouàs, Eivissa, Balearic Islands, Spain (I1, −100 to −120 cm)	Late Pleistocene to Holocene	distal right tibiotarsus	Mediterranean Institute for Advanced Studies
PE16	IMEDEA 24235	Es Pouàs, Eivissa, Balearic Islands, Spain (A2, −200 cm)	Late Pleistocene to Holocene probably Late Pleistocene	left femur (juvenile)	Mediterranean Institute for Advanced Studies
PE18	Sc10 US305 E15 no 60	Grotte Tastet, Pyrénées-Atlantiques, France	Late Pleistocene	right humerus shaft	Pierre Tastet (private owner)
PE19	B II	Le Morin, Gironde, France	Late Pleistocene	proximal right femur	Musée d'Aquitaine
PE26	X50383.1	Burwell Fen [skeleton collected in 1901]^a^	Mesolithic to Bronze Age	left humerus	Sedgwick Museum, University of Cambridge
PE27	X50284.2	Burwell Fen [skeleton collected in 1912]^a^	Mesolithic to Bronze Age	right tibiotarsus	Sedgwick Museum, University of Cambridge
PE28	X50385.1	Cambridge Fen [no date]^a^	Mesolithic to Bronze Age	left humerus	Sedgwick Museum, University of Cambridge
PE29	X50386.1	Burwell Fen [skeleton collected in 1890]^a^	Mesolithic to Bronze Age	left humerus	Sedgwick Museum, University of Cambridge
PE30	Pe 89/G IV/Bef 1.1/293-296	Pestenacker	Neolithic	radius	SNSB-Staatssammlung für Paläoanatomie München
PE31	Pe 90/I III/Bef 1.1 Fläche	Pestenacker	Neolithic	left phalanx manus	SNSB-Staatssammlung für Paläoanatomie München
PE32	88/17, oFZ	Xanten	Roman	left femur	LVR-Archäologischer Park Xanten-LVR-RömerMuseum
PE33	88/17, 28892 (6)	Xanten	Roman	left humerus	LVR-Archäologischer Park Xanten-LVR-RömerMuseum
PE40	84-43H	Caerleon, Wales	Roman	left humerus from near complete skeleton	National Roman Legion Museum
PE41	6109	Flixborough, Lincolnshire, England	Middle Saxon	proximal right tarsometatarsus	North Lincolnshire Museum
PE42	3891B	Flixborough, Lincolnshire, England	Middle Saxon	proximal right ulna	North Lincolnshire Museum
PE43	3891A	Flixborough, Lincolnshire, England	Middle Saxon	right ulna	North Lincolnshire Museum
PE44	6108	Flixborough, Lincolnshire, England	Middle Saxon	right tarsometatarsus	North Lincolnshire Museum
PE45	6247 (6347?)	Flixborough, Lincolnshire, England	Middle Saxon	proximal left humerus	North Lincolnshire Museum
PE46	5987	Flixborough, Lincolnshire, England	Middle Saxon	left tarsometatarsus	North Lincolnshire Museum
PE47	4197	Flixborough, Lincolnshire, England	Middle Saxon	right tarsometatarsus	North Lincolnshire Museum
PE48	6250 (6350?)	Flixborough, Lincolnshire, England	Middle Saxon	proximal right humerus	North Lincolnshire Museum
PE49	9697	Flixborough, Lincolnshire, England	Middle Saxon	right tarsometatarsus	North Lincolnshire Museum
PE50	4094	Flixborough, Lincolnshire, England	Middle Saxon	left tarsometatarsus	North Lincolnshire Museum

^a^ Dates that link these specimens to published data in [33]. Skeleton collected in 1901 is recorded in [33] as collected in 1907.

Sexual dimorphism in cranes has had limited consideration with regard to the interpretation of large cranes in the Late Quaternary of the Western Palaearctic—particularly with regard to the identification of *G. primigenia* [31,33]. Extant male *G. grus* are significantly larger than females [55], a factor that may have contributed to the misidentification of larger specimens as *G. primigenia* [8]. If *G. grus* were larger in the past, then it follows that remains of the largest individuals are likely to be from males, while those from smaller individuals (predominantly the size of modern male *G. gru*s) would be those from females. The relative lack of smaller individuals in the past (i.e. equivalent in size to modern female cranes) is consistent with this assumption [31,33]. This contrasts with the interpretation of Northcote [27] who believed that two different-sized crane species (*G. grus* and *A. antigone*) were living in sympatry in the past, although she later considered the large cranes to belong to *G. primigenia* [17,18]. The sympatry of the two different-sized cranes was taken as support for the existence of the additional larger extinct species and described as a non-intrinsic reason for the naming of extinct species. However, if the taxon in question is sexually dimorphic, taxonomic assignment of such different-sized specimens becomes more problematic [31,33]. It can also be the case that more than one subspecies of a bird can coexist in a region. This occurs, for example, in the case of the two different-sized subspecies of great cormorant, *Phalacrocorax carbo carbo* and *Phalacrocorax carbo sinensis*, found in Western Europe in recent times, although the smaller *P. c. sinensis* appears to have replaced the nominate subspecies over much of its range [61]. Perhaps a more appropriate example of co-occurring subspecies is the claim by Wetmore [62] for fossil grey crane *Antigone canadensis* remains in Florida, where large specimens belonged to either *A. canadensis pratensis* or *A. c. tabida* and smaller crane fossils belonged to either *A. c. canadensis* or *A. c. nesiotes*. Although *A. c. pratensis* is a Florida resident today, Wetmore [62] could not be sure which modern subspecies had existed in the past because the two modern subspecies in each size grouping are inseparable osteologically. He therefore suggested that an analogous situation had existed in the past where two differently sized unnamed subspecies had been sympatric when migrating during the Pleistocene [62].

More recently, the taxonomic identity of fossil remains has been resolved using ancient DNA (aDNA) methodologies (e.g. [63–67]). In some cases, fossils that appeared morphologically similar to modern species were, in fact, genetically distinct, while, in other cases, morphologically distinct fossils were found to be genetic matches to equivalent modern taxa. An example of this is shrews in the Late Pleistocene of Western Europe that were similar in size to the local modern shrew *Sorex* cf. *coronatus* but which was identified using aDNA methods as *Sorex tundrensis*, a species found today in Siberia [68]. Meanwhile, the extinct large shrew *Sorex macrognathus*, in the same range, was identified as *Sorex araneus*, albeit a large ecomorph from a distinct genetic population [68].

To resolve the taxonomic identity of G. *primigenia*, we sourced samples of cranes from the Late Quaternary of Europe. In order to test the different published hypotheses, we specifically sourced specimens that were larger than modern *G. grus* as well as ones that were consistent in size with *G. grus*. These samples cover the geographic range from which the putative extinct species *G. primigenia* has been identified [31,33,41,42]. Similarly, the temporal range of the sampled cranes covers much of the time from which *G. primigenia* has been identified. We conducted two analyses. The first was to phylogenetically identify the samples to species using aDNA. This provided a test for whether *G. primigenia* is a genuine species or if it belongs to one of the extant crane species. Second, the samples that were complete enough to be measured were used to correlate sex (based on molecular data) and size—that is whether the larger individuals (bigger than modern *G. grus*) represent males and the smaller individuals (generally the size of modern males) included females.

## 2. Material and methods

The *Grus* samples came from a range of localities in Europe from Britain to Spain, France and Germany and included specimens from sites dated to the Late Pleistocene (Grotte Tastet and Le Morin, France), Late Pleistocene/Early Holocene (Es Pouàs, Sant Antoni de Portmany, Eivissa, Balearic Islands, Spain), as well as Holocene (Pestenacker, Bavaria and Xanten Brunnen, North Rhine-Westphalia both in Germany; Flixborough in North Lincolnshire, as well as Caerleon in South Wales and the Cambridgeshire Fens in the UK) (table 2; figure 1). The ages of the specimens sampled are expressed in thousands of years calibrated before present (kyr cal BP) for the ages of samples associated with carbon dates; all other ages, not associated with carbon dates, are expressed as thousands of years before present (kyr BP) or years before present (yr BP). Otherwise, ages of samples are described using archaeological terms, such as Neolithic (5950–4450 yr BP in the UK) or Saxon (1300–884 yr BP in the UK), and we have given the age ranges on first mention of these terms for ease of comparison.

The specimens sampled include *Grus* bones that are larger than *G. grus* as well as ones that conform in size to modern *G. grus* (table 3). We especially targeted material from Late Pleistocene France in order to cover material similar to the type specimen described by Milne-Edwards [10]. The actual type material housed in the Muséum national d'Histoire naturelle in Paris is understandably unavailable for study since this involves destructive sampling.

**Table 3.** Measurements of the Quaternary *Grus* remains and the modern male and female *G. grus*.

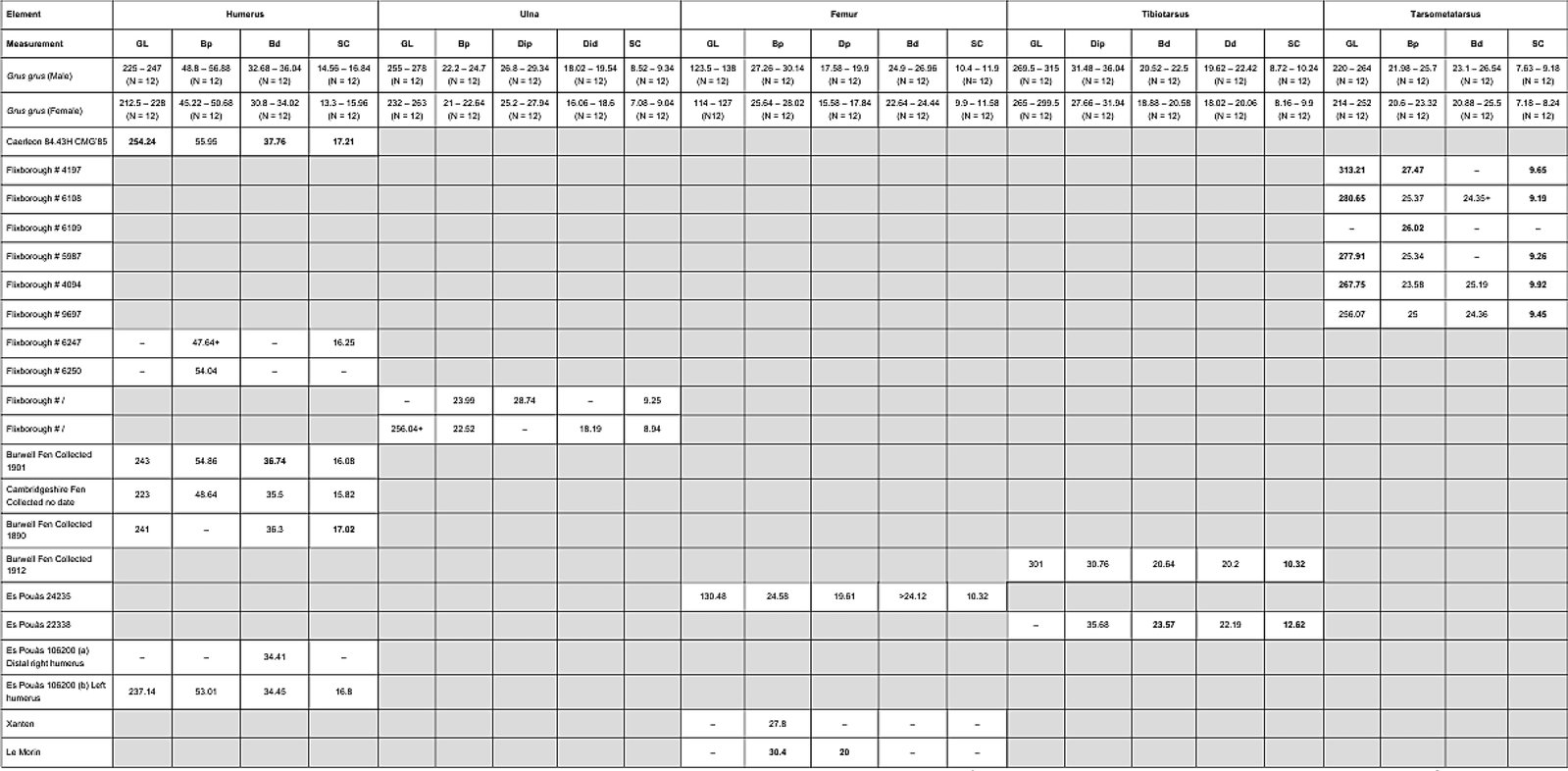

The modern measurements came from [33]. The measurements that exceed all modern common cranes, *G. grus*, are in bold. Measurements include GL, greatest length; Bp, proximal breadth; Dp, proximal depth; Dip, proximal diagonal; Bd, distal breadth; Dd, distal depth; Did, distal diagonal; SC, shaft breadth.

### 2.1. The sites

Cambridgeshire Fens, England (Mesolithic to Bronze Age 11950–2750 yr BP) (four specimens sampled and successfully analysed: laboratory nos. PE26 – 29, table 2).

The crane fossils from the Cambridgeshire Fens sampled are housed in the Sedgwick Museum at Cambridge University. The material was presented to the university by a number of individuals around the turn of the twentieth century (1890–1907). The sampled bones were derived from crane skeletons that were excavated during the constructions of water courses, operations to retrieve peat for fuel, as well as during ploughing [69]. The locality from which three of the skeletons sampled came from is Burwell Fen in the Cambridgeshire Fens, while a fourth sample was a skeleton from the Cambridgeshire Fens without further details. These skeletons are part of the collection of nine skeletons housed in the Sedgwick Museum, Cambridge University [31,33]. Radiocarbon dating and pollen analysis suggest that the crane fossils from the Cambridgeshire Fens date from zone VII of the Holocene, which equates to the Mesolithic to Bronze Age (11 950–2750 yr BP) [26,56,69]. Beaver (*Castor fiber*) remains from Cambridgeshire Fens, without further details, have been recently dated to between 4418–4247 cal yr BP and 5210–5205 cal yr BP [70] which is consistent with the previously proposed age of the crane bones.

The crane remains from the Cambridgeshire Fens were identified by Northcote [26] as those of common cranes because they were significantly shorter than those of the sarus crane. Furthermore, common cranes are known to have bred in East Anglia in recent times [71] having been absent from the sixteenth century [72] and returning to breed during the early 1980s [73].

Caerleon, South Wales (early Roman in the UK: *ca* 1865–1850 yr BP) (one specimen sampled and successfully analysed: laboratory no. PE40, table 2).

A single crane bone was sampled, a left humerus (84–43H), belonging to a collection of 62 bones from at least four individuals, which were excavated from a well fill at the site of the Caerleon Roman Legionary Museum [29]. The crane remains had signs of butchery marks as well as tooth-marks suggesting they were part of the human diet at the site that had been subsequently gnawed by a small carnivore. The well in which the remains were found, Well 2, was uncovered during works to enlarge the museum buildings during excavations between 1983 and 1985 [74].

The crane remains in Well 2 appear to date to *ca* 1865–1850 yr BP owing to the associated finds in the well fill and relate to Phase II occupation of the site. These finds are components of other ostensibly high-status food remains that included wild boar, red and roe deer, coriander, grape and fig [74].

Flixborough, Lincolnshire, England (Anglo-Saxon 1300–884 yr BP) (10 specimens sampled and successfully analysed: laboratory nos. PE41–50, table 2).

The Anglo-Saxon site of Flixborough in North Lincolnshire overlooks the floodplain of the River Trent and appears to have been a high-status rural (perhaps royal) settlement occupied from the late seventh to early eleventh centuries [75]. The site was excavated between 1989 and 1991 and yielded a very large assemblage of animal bones (41 206 identified specimens) dominated by domesticates. The bird bone assemblage included 11 601 identifiable fragments representing *circa* 27 avian taxa—the majority of remains being from chickens (5691) and geese (4557). Numerous remains of cranes (229) were present in all chronological phases [35].

The large assemblage of goose remains from the site were included in a successful early ancient mitochondrial DNA (MtaDNA) study that attempted to differentiate overlapping size categories of morphologically similar black *Branta* and grey *Anser* species [76]. Subsequently, the first MtaDNA study of cranes (which included large individuals) was conducted on *Grus* specimens from the site [77]. While the results demonstrated the likely presence of common cranes, *G. grus*, the results were not formally published, being a component of a PhD thesis [77]. Dobney *et al.* [35] suggested that the cranes from Flixborough likely represented a breeding population hunted locally in the Lincolnshire Fens—a reasonable assumption given the proximity of the two localities.

Grotte Tastet, Sainte-Colome, Pyrénées-Atlantiques, France (Upper Pleistocene, Late Middle Magdalenian 17–17.5 kyr cal BP) (one specimen sampled and successfully analysed: laboratory no. PE18, table 2)

Tastet cave is located in the Arudy Basin in the northwestern Pyrenees. The site has been excavated since 2012 by J.-M. Pétillon and provides an important archaeological assemblage attributed to the Late Middle Magdalenian [78,79] which is currently being studied by a multidisciplinary team. Several radiocarbon dates centred around 17–17.5 kyr cal BP have been done on ungulate remains, including horse and reindeer [45]. The inside of the cave has yielded several large crane remains (a fragmentary humerus found in several pieces, a right and left coracoid, a right scapula and a sternum containing tracheal rings) likely belonging to a single individual. Several of the bones have carnivore tooth-marks (punctures and pits) that may have introduced the carcass to the cave.

Le Morin, Pessac-sur-Dordogne, Gironde, France (Upper Pleistocene; Early Upper Magdalenian *ca* 16.5–15 kyr cal BP) (one specimen sampled and successfully analysed: laboratory no. PE19, table 2).

A crane proximal femur from Le Morin rock shelter is housed in the Musée d'Aquitaine in Bordeaux. The specimen comes from Unit B, level BII at the base of the sequence excavated by R. Deffarge at the end of the 1950s (see [80] for a historical background). A re-evaluation of the abundant archaeological material assigned Unit B to an early phase of the Upper Magdalenian which developed regionally between *ca* 16.5 and 15 kyr cal BP [80–82]. Unit A, above Unit B, is divided into four artificial levels and belongs mainly to the Late Upper Magdalenian (*ca* 15.5–14 kyr cal BP) with Azilian and Laborian elements, especially towards the top of the stratigraphy. The four radiocarbon dates on osseous industry and taxonomically identified animal bone remains from level BI are consistent with the regional chronology of the Early Upper Magdalenian. The single date obtained from BII, on a barbed point, gives a slightly more recent date although this level is stratigraphically below BI. Refits undertaken on fragments of faunal material from this old excavation show that most bone fragment associations were between specimens from the same level although some were between fragments found in different levels. This suggests that some movement of material had taken place between layers or that specimens were misattributed to layers.

The crane femur from Le Morin was identified as *G. primigenia* by Delpech [15] during a general analysis of the faunal remains. The femur contains medullary bone and has cut marks indicating that a female crane was processed by humans during the breeding season [44,47]. Other bird remains are represented by a very abundant collection of snowy owls (*Bubo scandiacus*) analysed in detail by Gourichon [83], followed by ptarmigan (*Lagopus* sp.), a few ducks (*Anas* sp.) and a swan (*Cygnus* sp.) [47,80].

Pestenacker, Lkr. Landsberg am Lech, Bavaria, Germany (Neolithic: 5446–5360 yr BP) (two specimens sampled and successfully analysed: laboratory nos. PE30–31, table 2).

The Late Neolithic site near the village Pestenacker is located in a tributary valley of the Lech River, south of the city of Augsburg. Excavated in two large-scale projects between 1988 and 1993 and again between 2000 and 2004, the site became part of the UNESCO World Heritage ‘Prehistoric pile dwellings around the Alps’ in 2011 [53,84]. The timbers used for the buildings were waterlogged and therefore exceptionally well preserved. This allowed the settlement to be accurately dated using dendrochronology to between 5446 and 5360 yr BP. Archaeologically, the site belongs to the Late Neolithic Altheim Culture.

The fauna from the first excavation project was analysed and published by Vagedes [85]. The crane bones were identified as common crane owing to their size. Although not very frequent, bones of cranes are found consistently in southern German archaeological sites dating from the Holocene period until the early modern era [86]. Until the late nineteenth century, cranes regularly bred in the wetlands of southern Bavaria [87].

Xanten, Kr. Wesel, North Rhine-Westphalia, Germany (Roman: 1850–1825 yr BP) (two specimens sampled and successfully analysed: laboratory nos. PE32–33, table 2).

Xanten is situated in the Lower Rhine Valley on the left bank of the river. The Roman occupation began around 1963 yr BP with the first military camp Vetera Castra, south of the modern town. The first civilian settlement dates to around 1940 yr BP and was replaced by the Colonia Ulpia Traiana (CUT), founded under Emperor Traian around 1850 yr BP [88,89]. The town became a major centre in the province of Germania Inferior, which was finally abandoned in the late fourth century.

The crane remains were excavated from a well dating to the first quarter of the second century [90]. The fauna was identified and published by Pöllath & von den Driesch [52] and situated in the Rhine lowlands; the high diversity of the aquatic fauna was not surprising. Crane remains are rare in prehistoric settlements along the Lower Rhine Valley but more frequently found at sites dating from the Roman era to early modern times [86].

Es Pouàs, Eivissa, Balearic Islands, Spain (Upper Pleistocene and Holocene layers 36.7–34.5 cal kyr BP to 2.3–2.1 cal kyr BP) (three specimens sampled and successfully analysed: laboratory nos. PE14–16, table 2).

The fossil site of Es Pouàs consists of a sinkhole that acted as a sedimentary trap over the last 30 000 years, gradually filling with a mixture of fine clastic deposits, composed of a clay matrix containing calcareous cave-rock debris ranging from very small grains to small pebbles and abundant fossils [50,91].

Between 1989 and 1994, Alcover and collaborators carried out a total of six months of fieldwork at this site, excavating less than 10% of the total sedimentary volume. These deposits include levels from the Upper Pleistocene–Holocene, spanning from 36.7–34.5 cal kyr BP to the earliest evidence of human presence (to 2.3–2.1 cal kyr BP). They yielded an exceptionally large assemblage of material estimated to exceed 120 000 bones (with approx. 30 000 catalogued), curated at the Institut Mediterrani d'Estudis Avançats (IMEDEA). Most of these fossils were mainly accumulated through the activity of predatory birds such as *Haliaeetus albicilla*, kestrel *Falco tinnunculus* and Eurasian scops owl *Otus scops*, among others [92]. The analyses of the terrestrial vertebrate assemblage revealed more than 100 bird species, 5 or 6 bat species, a lizard (*Podarcis pityusensis*) and a snake (*Vipera latastei ebusitana*), with no evidence of terrestrial mammals within this stratigraphic sequence [49,50,92–94].

The palaeoenvironment inferred from the avian community of Es Pouàs suggests a variable climate, colder in the lower layers and warmer in the upper ones. The landscape was characterized by open plains, rocky areas, and zones of forest and scrubland, with a humid wetland in the vicinity [50].

A recent review of the large non-passerine avifauna from the site led to the identification of more than 400 crane bones, corresponding to at least 22 individuals (based on the first pedal phalange of the second left toe) belonging to both adult and juvenile specimens (ranging from neonates to fully developed individuals). The samples taken for the present study fall within the Late Pleistocene and Early Holocene (table 2).

### 2.2. Morphometric analysis

All skeletal remains of *Grus* sampled, which were complete enough, were measured so that they could be compared with the corresponding bones of modern common cranes. The skeletal elements sampled that were measured include humeri, ulnae, femora, tibiotarsi and tarsometatarsi (table 2). The measurements taken are described in detail in [33] and were taken using digital calipers. Abbreviations of the measurements (taken from [33]) are as follows: GL, greatest length; Bp, proximal breadth; Bd, distal breadth; SC, shaft breadth; Dip (DP in the ulna [33]), proximal diagonal depth; Did, distal diagonal depth; Dp, proximal depth; Dd, distal depth. Further details of measurements are detailed in [33]. The measurements of common cranes with which the sampled remains are compared also come from Stewart [33].

### 2.3. Extraction of ancient DNA, library preparation and whole-genome sequencing

Laboratory procedures were completed in a state-of-the-art facility for ancient DNA (aDNA) extraction at the Centre of Palaeogenetics (a joint facility for the Swedish Museum of Natural History and Stockholm University) dedicated to working on aDNA. We obtained approximately 50 mg of bone powder from each specimen using a Dremel high-speed multi-tool, and DNA extraction was done according to the protocol in Dabney *et al.* [95]. The extraction was eluted in 50 µl TET (Tris–EDTA + 0.05% Tween). We then built double-stranded Illumina libraries according to Meyer & Kircher [96] using 20 µl of the DNA extract that was also treated with USER enzyme to excise uracil residues resulting from post-mortem damage [97,98]. For blunt end repair, the DNA was incubated with 6U USER enzyme for 3 h at 37°C, followed by the addition of 0.8 µl T4 DNA polymerase (Thermo Scientific), and incubation at 25°C for 15 min and 12°C for 5 min and was then cleaned using MinElute (QIAGEN, Hilden, Germany) spin columns and eluted in 20 µl EB Buffer following the manufacturer's protocol. Next, we performed an adapter ligation step where DNA fragments of the libraries were ligated to a combination of incomplete, partially double-stranded P5 and P7 adapters. This reaction was performed in a 40 µl reaction volume using 20 µl of blunted DNA from the clean-up step and 2 µl P5–P7 adapter mix (0.5 µM final concentration for each adapter) per sample with a final concentration of 1 × T4 DNA ligase buffer, 5% PEG-4000, 5U T4 DNA ligase (Thermo Scientific). The samples were incubated for 30 min at room temperature and cleaned using MinElute spin columns as described above. Next, we performed an adapter fill-in reaction in 40 µl final volume using 20 µl adapter-ligated DNA with a final concentration of 1× ThermoPol Reaction Buffer, 250 µM of each dNTP, 12U Bst Polymerase (Thermo Scientific). The libraries were incubated at 37°C for 20 min and heat-inactivated at 80°C for 20 min.

The libraries were then amplified with double-indexed P5–P7 primers. The amplification was performed in 25 µl volumes with 3 µl of adapter-ligated library as template, with the following final concentrations: 1× AccuPrime reaction mix, 0.3 µM P7–P5 indexing primer mix and 7 U AccuPrime Pfx (a high fidelity polymerase, Thermo Scientific), and the following cycling protocol: 95°C for 2 min, 12 cycles at 95°C for 30 s, 55°C for 30 s, 72°C for 1 min and a final extension at 72°C for 5 min. Purification and size selection of the amplified and indexed libraries were then performed using Agencourt AMPure XP beads (Beckman Coulter, Brea, CA, USA), first using 0.5× bead:DNA ratio and secondly 1.8× to remove long and short (i.e. adapter dimers) fragments, respectively. Library concentration was measured with a high-sensitivity DNA chip on a Bioanalyzer 2100 (Agilent, Santa Clara, CA, USA). Finally, the libraries were sequenced on Illumina NovaSeq 6000 and NovaSeq X Plus flowcells with a 2 × 151 bp (base pairs) set-up, including Xp kit (validated method) at the Science for Life Laboratory (National Genomics Institute, Stockholm). In total, we obtained 45 Gb data (1488 million reads) for the fossils.

### 2.4. Trimming of sequencing reads, initial reference mapping and phylogenetic reconstruction

The Illumina sequencing reads from the fossils were processed through a custom bioinformatic pipeline (github.com/mozesblom) to eliminate adapter contamination, low-quality bases and low-complexity sequences. This involved merging overlapping read pairs with PEAR [99], removing PCR duplicates using SuperDeduper [100] and performing quality trimming and adapter removal with Trimmomatic v.0.32 [101] under default parameters. The resulting trimmed reads were then mapped to the mitochondrial genome of *G. grus* (NC_020577) [102] using BWA (v.0.7.18 [103]) aln with parameters suitable for ancient DNA (−l 1024 −n 0.01 −o 2). We removed fragments shorter than 30 bp DNA using SAMtools (v.15.1 [104]) view −e ‘length(seq)>29.’.

Phylogenetic inferences were conducted using maximum-likelihood analysis in MEGA11 [105]. First, to assess the species allocation of the fossil remains, we constructed a dataset from their nearly complete mitochondrial genomes (excluding the control region), alongside the following sequences downloaded from GenBank: *A. antigone* (NC_020581), *Grus carunculatus* (FJ769843), *G. grus* (NC_020577), *L. leucogeranus* (NC_020574) and *Grus virgo* (FJ769845). This alignment had a total length of 15 667 bp. When analysing this dataset, we applied the general time reversible model for nucleotide substitutions with the following parameters: Ts/Tv 8.8271, gamma shape 1.1816, invariant 0.5979, r_AC_ 0.1390, r_AG_ 0.4093, r_AT_ 0.7420, r_CG_ 1.2318, r_CT_ 2.4778 and r_GT_ 1.0. Subsequently, to investigate relationships among the fossils themselves, a separate analysis was performed using a dataset of the control region alone (alignment length: 1021 bp). Owing to alignment difficulties with distantly related taxa, this second analysis included only *A. antigone* and *G. virgo* as out-groups. This dataset was also analysed using the general time reversible model for nucleotide substitutions. The following parameters were used: Ts/Tv 2.7703, gamma shape 16.2788, invariant 0.4277, r_AC_ 0.6796, r_AG_ 0.8556, r_AT_ 0.9800, r_CG_ 1.1163, r_CT_ 1.3684 and r_GT_ 1.000. In both analyses, the robustness of all phylogenetic nodes was assessed through parametric bootstrapping (1000 replicates).

### 2.5. DNA-based sexing

We estimated the sex of each fossil specimen using the SeXY pipeline [106], which is based on the principle that avian females (ZW) possess half the gene dosage of the Z chromosome compared to males (ZZ). First, we extracted the Z sex chromosome and a similarly sized autosome (chromosome 4) from the nuclear genome of *G. grus* (assembly GCA_964106855). Trimmed reads were mapped against these two reference chromosomes using BWA aln (v.0.7.18 [103]). Mean coverage depth was then calculated across 10 million randomly sampled sites on each chromosome using SAMtools depth (v.15.1 [104]). We computed the ratio of mean coverage on the Z chromosome to that on chromosome 4 (the Z:A ratio). This entire process, random site sampling, coverage calculation and ratio estimation, was repeated 10 times. Final sex assignments were made based on the mean Z:A ratio and its standard deviation across iterations.

## 3. Results

### 3.1. Morphometric analysis

The *Grus* samples analysed that could be measured and compared with those of modern common cranes are presented in table 3.

The left humerus from Caerleon (PE40) is larger than 24 (12 males and 12 females) modern common cranes *G. grus* measured in three of four measurements, including greatest length (GL), distal breadth (Bd) and shaft breadth (SC) and excluding proximal breadth (Bp) which conforms to that of a modern male (tables 3 and 4).

**Table 4.** Crane remains sexed by genetics and measurements.

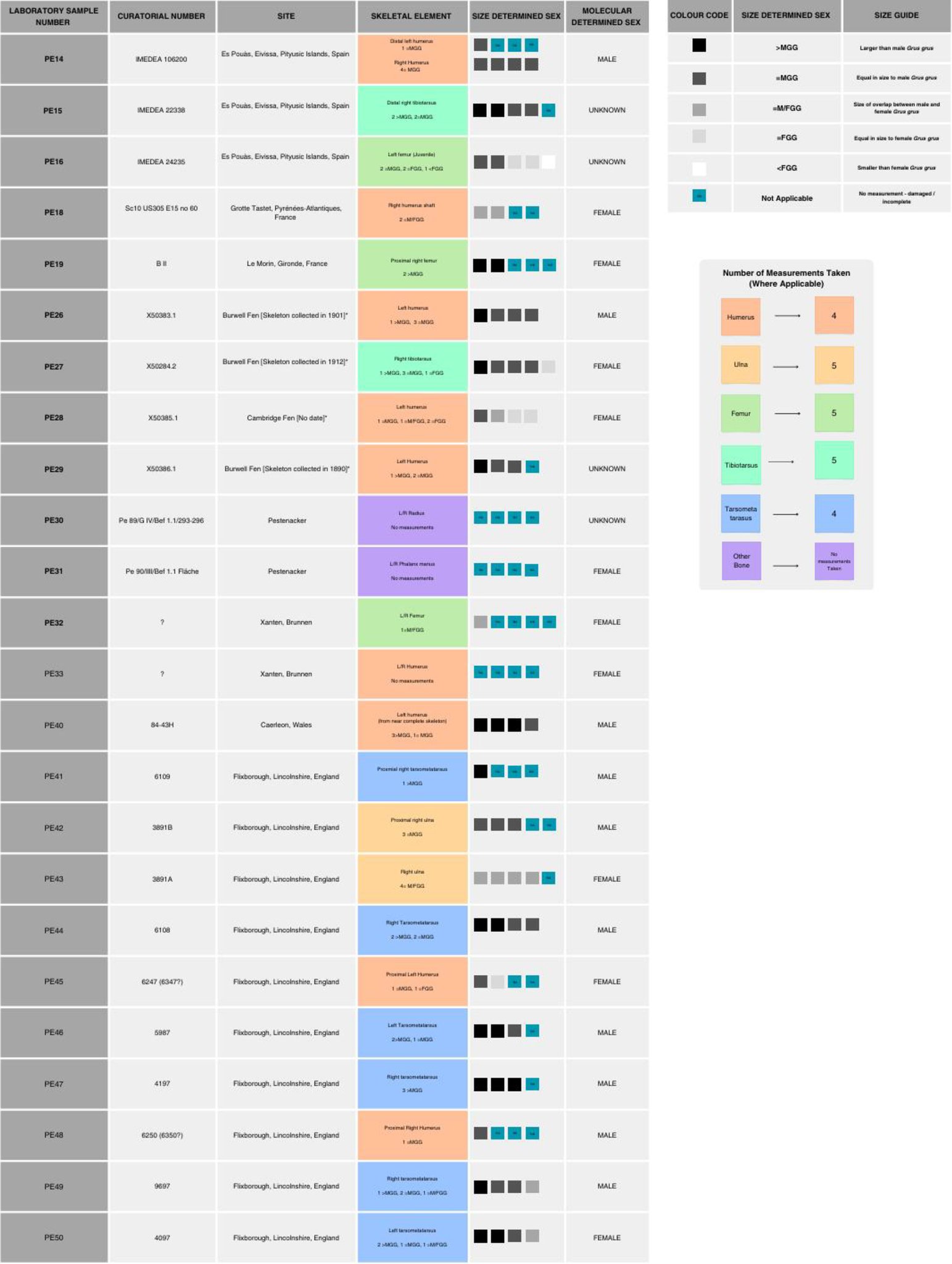

The sexes determined by genetics are those presented in figure 3. Each of the measurements of the ancient crane remains is presented relative to modern sexed common crane—larger than male common crane >MGG, the size of modern common crane = MGG, in the overlap size range between male and female common crane = M/FGG, the size of female common crane = FGG and smaller than common crane <FGG.

The Flixborough samples include one proximal right humerus, one proximal left humerus, one right ulna, one proximal right ulna, three right tarsometatarsi, one proximal right tarsometatarsus and two left tarsometatarsi (table 2). Of these four out of six of the tarsometatarsi (4197—PE47, 6108—PE44, 5987—PE46 and 4094—PE50) have at least two measurements that exceed all modern *G. grus* with which they were compared, and the two remaining specimens (6109—PE41 and 9697—PE49) had a larger shaft breadth than all modern measurements (tables 3 and 4). Most other measurements on the remaining specimens were consistent with modern male *G. grus*, and two specimens had measurements that fell in the overlap in measurements between the males and females (tables 3 and 4). The two humeri sampled (6247—PE45 and 6250—PE48) had measurements in the range of modern males or females (tables 3 and 4). The two ulnae (proximal right ulna 3891B—PE42; complete right ulna 3891A—PE43) differed in size with the larger one having measurements the size of modern males, while the smaller one had measurements in the overlap between male and female measurements (tables 3 and 4).

The Cambridgeshire Fen specimens include three humeri, two of which (collected in 1901, PE26; and 1890, PE29) with one measurement each (Bd and SC, respectively) being larger than the size of modern males and all other measurements are the size of modern males (tables 3 and 4). The other humerus (one with no date—PE28) is smaller with two measurements (GL and Bp), the size of modern females, one (Bd) the size of modern males and a further one (SC) in the overlap range between the modern sexes (tables 3 and 4). The tibiotarsus from the Fens meanwhile has one measurement larger than that of all cranes today (SC) and three measurements the size of males (GL, Bd and Dd) with a final measurement (Dip) in the modern female range (tables 3 and 4).

Of the three specimens from Es Pouàs, there is a left femur (24235—PE16) which appears to belong to a juvenile which may account for one of the measurements (Bp) being smaller than all modern common crane measurements (tables 3 and 4). The other measurements of the specimen include two (Bd and SC) that lie in the range of modern females and two (GL and Dp) that are within the male common crane range (tables 3 and 4). The distal tibiotarsus (22338—PE15) has two measurements (Bd and SC) above the range of modern males and two (Dip and Dd) that fall within the range of modern common cranes (tables 3 and 4). Finally, the distal right humerus (106200a—PE14) has a measurement the size of modern males, and the associated left humerus has all four measurements (GL, Bp, Bd and SC) in that range (106200b in tables 3 and 4).

Of the German and French material, only two could be compared with modern measurements, the proximal femora from Xanten and Le Morin. The Xanten femur (PE32) had a single measurement (Bp) that fell in the overlap range of the modern male and female *G. grus* (tables 3 and 4). Meanwhile, the two measurements on the Le Morin proximal right femur (Bp and Dp) (PE 19) were larger than the modern measurements (tables 3 and 4). It should be noted that the right humerus from Tastet (Sainte-Colome) was not complete enough for measurements although an associated right coracoid had dimensions (medial length LM >65 mm and shaft breadth SC 16.2 mm) that fell in the overlap range of modern male and female *G. grus* shown in [33].

#### 3.1.1. Mitogenomic sequencing and phylogenetic analysis

Most samples were sequenced to a low depth of coverage, as the primary objective of the mitogenomic sequencing was species assignment. For some samples, this low coverage, combined with a considerable DNA degradation, resulted in high proportions of missing data (supplementary material, table S1). Nevertheless, phylogenetic analysis of an alignment consisting of 15 667 bp of the mitochondrial genome confirms that all sampled *Grus* fossils from Western Europe belong to *G. grus*. This assignment includes morphologically larger specimens, demonstrating that they do not represent an extinct *Grus* species or any other extant large Palaearctic crane (figure 2).

We included the Siberian crane (*G. leucogeranus*) and the sarus crane (*G. antigone*) in our analysis, as these species have been suggested as possible identities for some large European crane fossils (e.g. [19–21,24]). Consistent with Krajewski *et al.* [102], our results place both species as distantly related to the common crane within the Gruidae clade, thereby excluding them as candidates for the sampled remains.

Phylogenetic analysis of the mitochondrial control region, while consistent with a *G. grus* designation, did not resolve relationships among the fossils, as indicated by a lack of bootstrap support for internal nodes (supplementary material, figure S1).

#### 3.1.2. Sex estimation

Molecular sex estimation was possible for 22 of the fossil specimens. Twenty of these were confidently assigned as either male or female based on their Z:A coverage ratio, in accordance with the classification thresholds of Cabrera *et al.* [106] (supplementary material, tables 4 and S1). However, two specimens yielded a Z:A ratio between 0.7 and 0.8, which falls within the authors’ designated ‘uncertainty zone’, precluding a definitive sex assignment.

## 4. Discussion

The large cranes of the later Quaternary of Europe were described as an extinct species in the nineteenth century [10,11]. They have received more attention in the scientific literature than any other European Quaternary Palaearctic bird species since that time with various differing interpretations. The following hypotheses have been proposed for these large cranes:

**Hypothesis 1**

– They belong to the extinct large crane species *G. primigenia* [10–18].

**Hypothesis 2**

– They belong to the Asian sarus crane (*A. antigone*) [19–27].

**Hypothesis 3**

– Some Late Pleistocene specimens belong to the Siberian white crane (*L. leucogeranus*) [31,33,36].

**Hypothesis 4**

– They are larger common cranes, *G. grus* [28–35].

**Hypothesis 5**

– They belong to an extinct subspecies of common crane, *G. grus*, found only in the British Isles [31,33,35].

The phylogenetic analyses based on a 15 667 bp mitochondrial genome alignment and on an alignment of the mitochondrial control region (1021 bp) conclusively demonstrate that all the ancient remains analysed during this study that yielded viable aDNA belong to *G. grus* (figure 2 and supplementary material, figure S1). The phylogenetic analysis included the two other species of crane that had been suggested as possible identities for the large European cranes—that is, *A. antigone* and *L. leucogeranus*. These species are clearly taxonomically distinct from the sampled large cranes (figure 2 and supplementary material, figure S1) and so confirm the rejection of hypotheses 2 and 3. Thus, hypotheses 1, 2 and 3 are rejected, while hypothesis 4 is confirmed. All 18 crane specimens analysed cluster with *G. grus* on the phylogenetic trees and show no sign of being distinct from that species to merit a separate species status (figure 2 and supplementary material, figure S1). Hypothesis 5 can also be rejected since samples from the British Isles (Flixborough, Caerleon and the Cambridgeshire Fens) do not form a distinct clade, instead being interspersed with individuals from the European continent. This also suggests that there was no phylogeographical structure in Late Quaternary west European *G. grus* based on the mitochondrial genome. There is also no chronological structure visible among the samples, as the later Holocene (Neolithic, Bronze Age, Roman and Middle Saxon) samples from Germany and Britain are interspersed with French Late Pleistocene and Spanish Late Pleistocene/Holocene specimens (figure 1).

The present study also used the ratio of mean coverage on the Z sex chromosome to that on the autosomal chromosome 4 (the Z:A ratio) (figure 3) to estimate what sex the different-sized specimens were. The purpose of this was to test whether the size of male and female Quaternary large cranes has changed. It seems likely that the size of the species has decreased over time since Saxon times and that the sexual dimorphism in the common crane appears to have resulted in the assumption that two cranes had existed in the past: a larger one that was attributed to *G. primigenia* and a smaller one that was thought to be *G. grus*.

**Figure 1.**
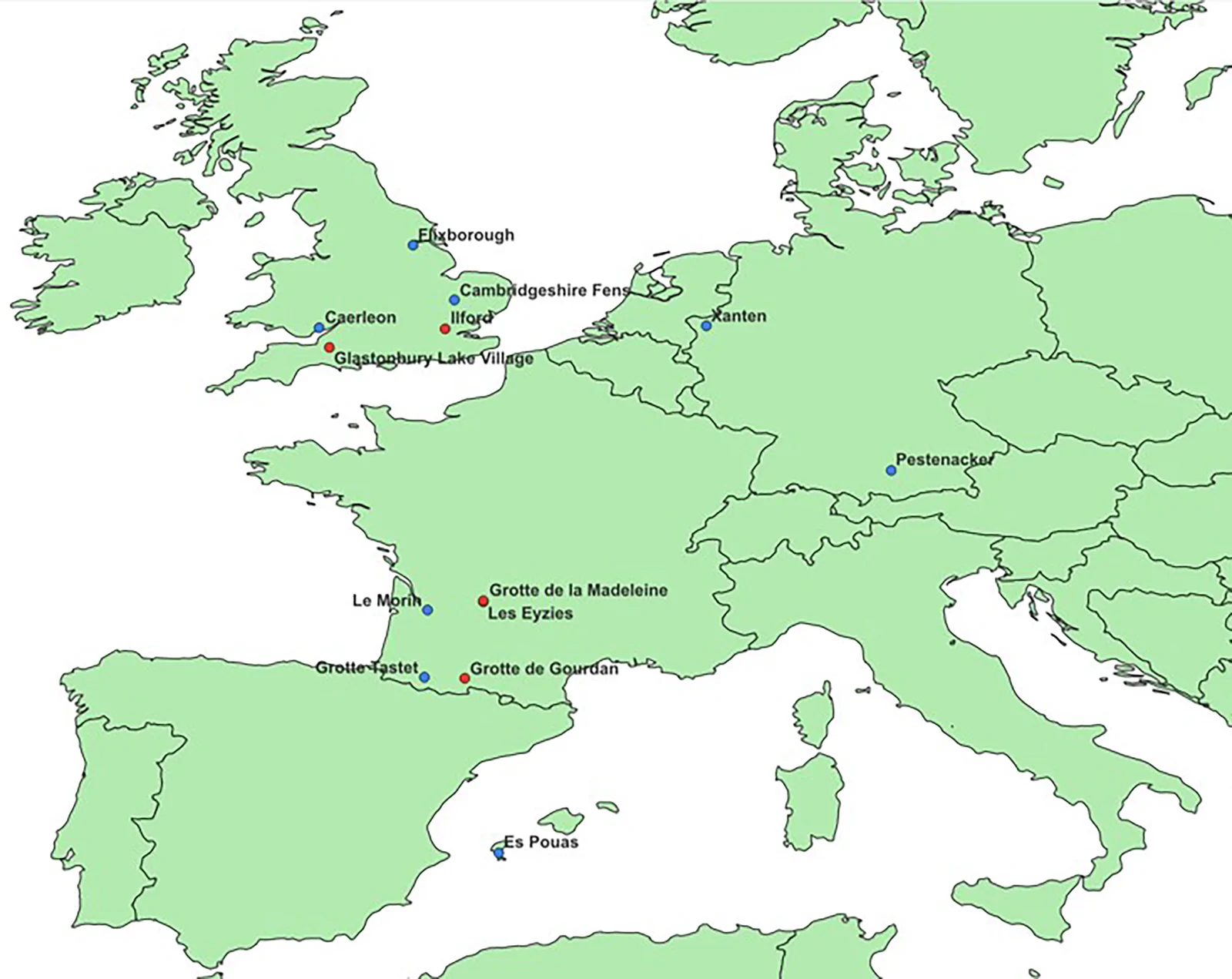
Map of sites with crane remains attributed to *Grus primigenia* or large *G. grus* mentioned in the text. The sites sampled in this study are represented by blue points, while the red points represent the sites mentioned in the text that were not sampled.

**Figure 2.**
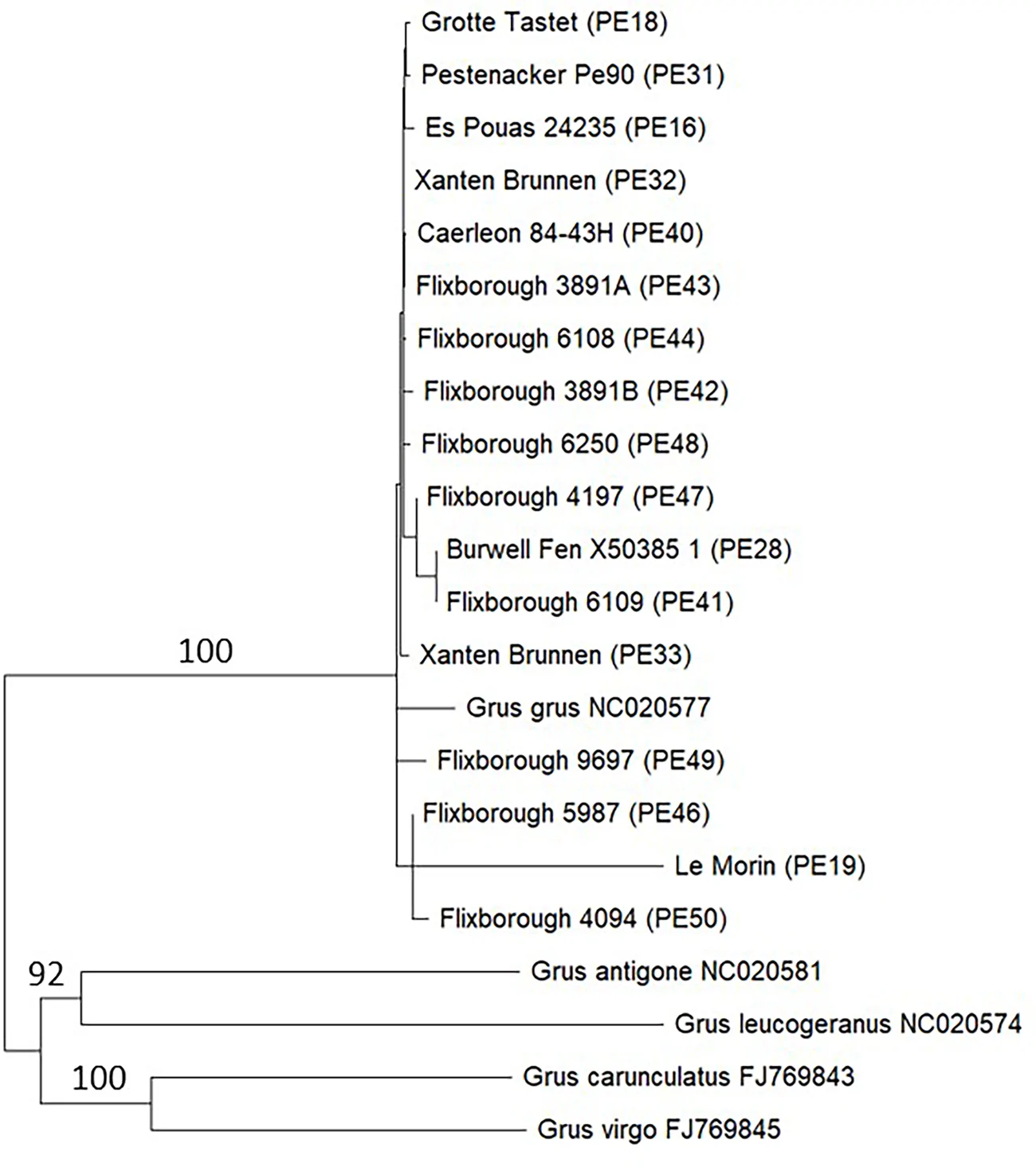
Maximum-likelihood analysis based on the partial mitochondrial genome, except the control region. The aligned dataset consists of 15 667 bp. The general time reversal (gamma distributed with invariant sites) model for nucleotide substitutions was used to estimate the tree (log likelihood for the best-fit tree = −34 490.53). Support values are based on 1000 bootstrap replicates.

**Figure 3.**
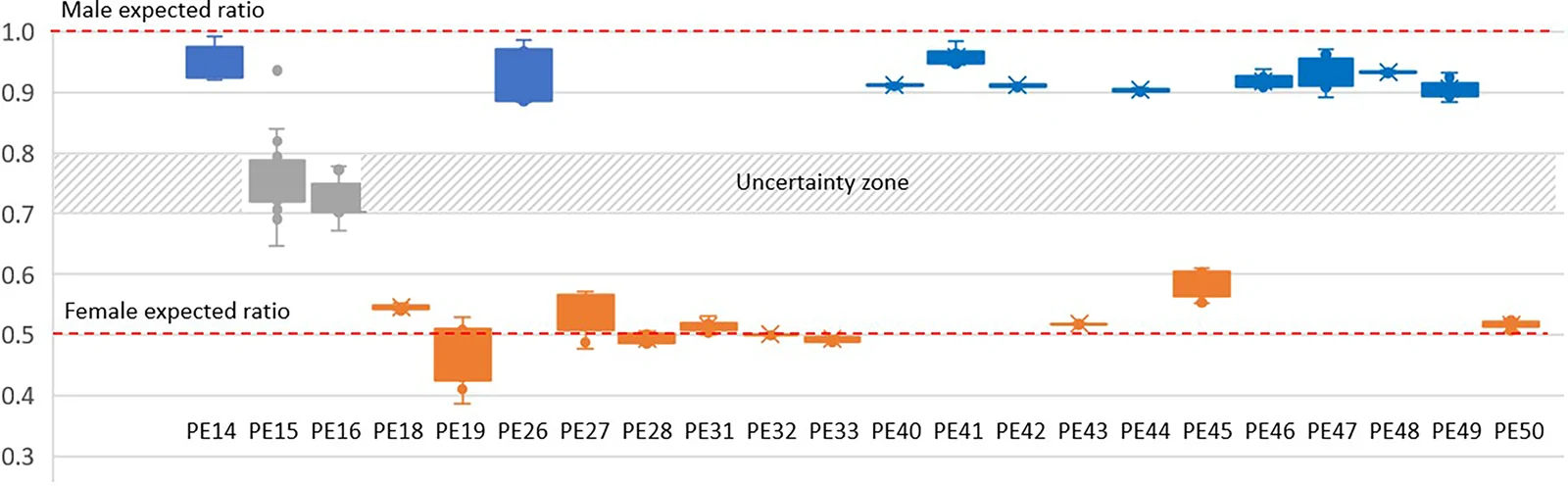
Genetic sex identification of fossil cranes based on the ratio of mean sequencing coverage on the Z chromosome to that on an autosome (chromosome 4), known as the Z:A ratio. According to the avian ZZ/ZW system, males (ZZ) are expected to have a ratio of approximately 1.0, while females (ZW) are expected to have a ratio of approximately 0.5. Error bars represent the standard deviation of the mean coverage calculated from 10 iterations of resampling 10 million random sites per chromosome. Following Cabrera *et al.* [106], individuals with Z:A ratios between 0.7 and 0.8 (grey shaded area) were classified as uncertain.

The results of the genetic sex determination of the ancient crane specimens demonstrate that the bones identified as males (from Flixborough, Caerleon, the Fens and Es Pouàs) included measurements that either exceeded or were equal to those of male common cranes today (figure 3; table 4). One measurement of a right tarsometatarsus from Flixborough (PE49) that was identified genetically as male had a single measurement (distal breadth) that fell into the overlap size range between males and females today (figure 3; table 4). The females meanwhile included measurements that were greater than those of modern males, were the size of males, fell into the overlap range between males and females or were the size of females (table 4). The range was, however, skewed with three measurements that fell into the modern female range, six in the male–female overlap range, five in the modern male range and three in the larger than modern male range (table 4). This suggests that females in the past were also larger on average than their modern counterparts. None of the ancient females had dimensions that solely agreed with that of modern female cranes, generally falling in the overlap range between modern male and female *G. grus*, or, if a measurement fell in the female crane range, the other measurements on that bone were larger (table 4). This is further confirmed by the previous large-scale morphometric analysis of past cranes in which few cranes from the past fell in the size range of modern female common cranes [33]. The distal femur from Le Morin (PE19) is larger than all modern specimens in both measurements and is identified genetically as female (table 4). This is consistent with the findings of medullary bone found inside femur [44] (PE27) (table 4).

The size change documented here in the common crane occurred within the Holocene rather than between the Pleistocene and Holocene as is often the case although this is not without precedent [8,33]. Furthermore, the results suggest that all the remains in this study belong to *G. grus* which, in turn, suggests that *G. primigenia* may not be a valid species. This includes material dated to the Late Pleistocene of southern France which is coeval with the type material so it seems likely that material would also prove to belong to *G. grus* if studied. Unfortunately, that material was not available for study, so a small amount of doubt remains and the extinct crane *G. primigenia* could still prove valid.

## 5. Conclusion

In conclusion, our data confirm that large Late Quaternary cranes of the Western Palaearctic belong to common crane and not to an extinct species or to any other extant large crane species such as the sarus or Siberian white crane. This suggests that the species *G. primigenia* may not be valid although until the type material is studied this cannot be confirmed with certainty. What is not known is why an apparent size reduction occurred in common crane after the eleventh century AD, the age of the youngest large cranes in Europe that we are aware of. It is tempting to conclude that the size change seen in the common crane is owing to climate change and/or human land use change such as wetland drainage, but for now this remains speculation. The fact that the mute swan appears to have undergone size reduction during the Holocene may provide support for this [107]. This study emphasizes that if claims such as that by Cooke *et al.* [7] that large numbers of extinct birds remain undiscovered are to be upheld, then detailed studies of individual species such as that presented here need to be conducted.

## Supplementary Material



## Data Availability

The raw sequences are available in the European Nucleotide Archive (ENA) under the study accession number PRJEB110935 (https://www.ebi.ac.uk/ena/browser/view/PRJEB110935). DNA alignments are available at https://github.com/perericson/DNA-study-of-fossil-cranes.git. Additional information about the dataset is found in the supplementary material [108].
