# Supplementary material for: Ancient DNA study resolves the taxonomic status of the extinct large Palaearctic crane Grus primigenia Milne-Edwards, 1871 (Aves: Gruiformes: Gruidae)
Source: R Soc Open Sci. 2026 Oct 28;13(10):260699. doi: 10.1098/rsos.260699 (PMC13714411; doi:10.1098/rsos.260699)
Supplement: Supplementary file 1 [file rsos.260699.zip › rsos.260699/rsos260699_si_001.pdf]

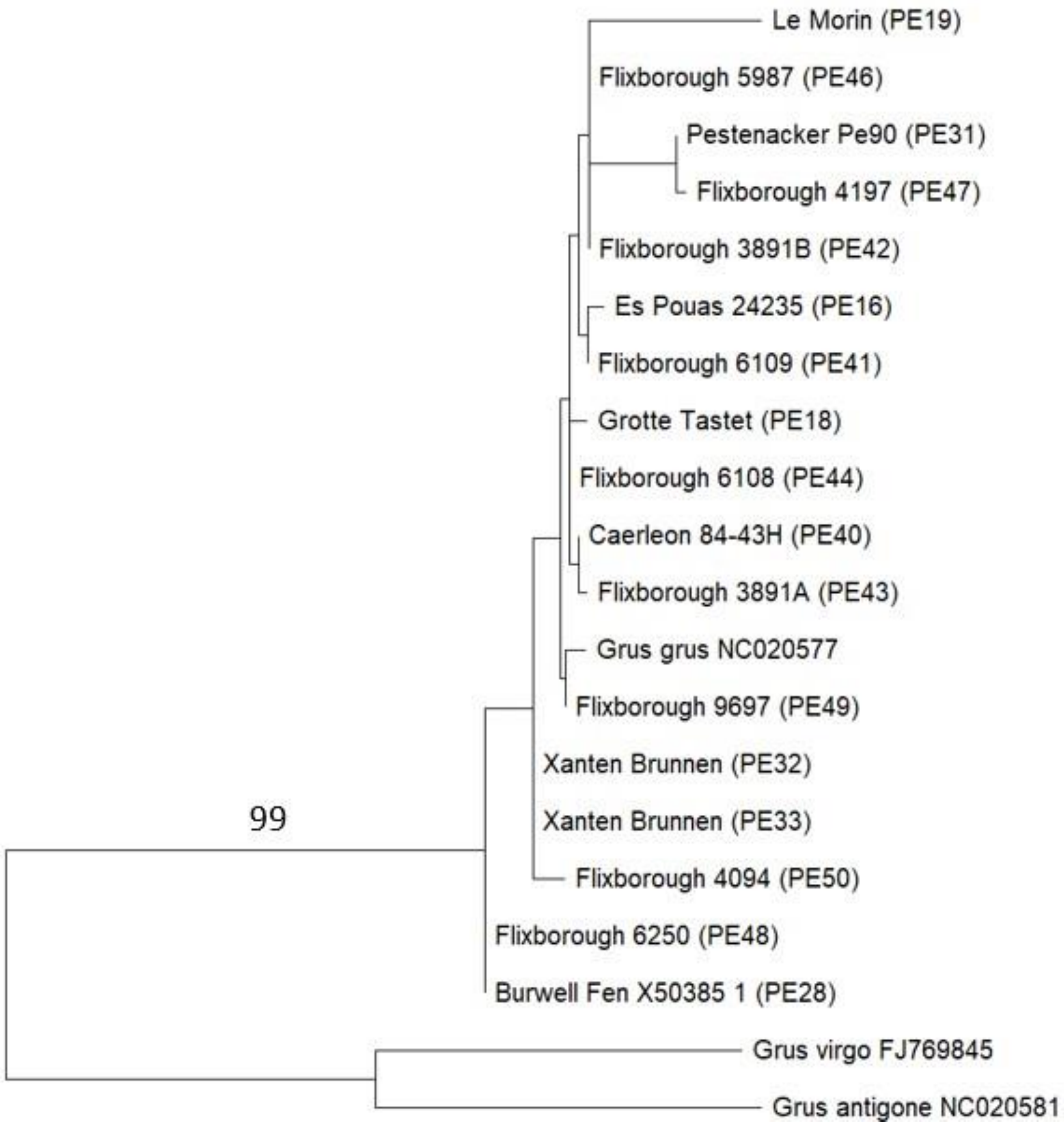

Figure S1. Maximum-likelihood phylogeny based on an alignment of the mitochondrial control region (1,021 bp). The tree was inferred under the General Time Reversible model with a gamma distribution and invariant sites (GTR+G+I). Nodal support is derived from 1000bootstrap replicates (only values >70 are shown). The log-likelihood of the best-fit tree was -2128.23.
