# Supplementary material for: Ancient DNA study resolves the taxonomic status of the extinct large Palaearctic crane Grus primigenia Milne-Edwards, 1871 (Aves: Gruiformes: Gruidae)
Source: R Soc Open Sci. 2026 Oct 28;13(10):260699. doi: 10.1098/rsos.260699 (PMC13714411; doi:10.1098/rsos.260699)
Supplement: Supplementary file 1 [file rsos.260699.zip › rsos.260699/rsos260699_si_003.docx]

**FURTHER CORRECTION TO STEWART ET AL:**

**Additional Acknowledgement:**

“The authors acknowledge support from the National Genomics Infrastructure in Stockholm funded by Science for Life Laboratory, the Knut and Alice Wallenberg Foundation and the Swedish Research Council, and NAISS/Uppsala Multidisciplinary Center for Advanced Computational Science for assistance with massively parallel sequencing. Computational resources were provided by the National Academic Infrastructure for Supercomputing in Sweden (NAISS) at the PDC Center for High Performance Computing, KTH Royal Institute of Technology, on the Dardel system.”

**Questions in your list:**

Q10: "Next, we performed an adapter fill-in reaction in 40 μl final volume using 20 μl adapter-ligated DNA with a final concentration of 1x ThermoPol Reaction Buffer, 250 μM of each dNTP, 12U Bst Polymerase (Thermo Scientific)."

Q15:

14. IS A THESIS SO MAY NEED TO BE CHANGES TO:

Delpech F. 1967 *Recherches paléontologiques concernant quelques gisements du Magdalénien VI: stations de la Gare de Couze (Dordogne), du Morin (Gironde) et de Duruthy (Landes)*. Thèse de doctorat de 3ème cycle, Faculté des Sciences de l'Université de Bordeaux, 2 volumes.

Q16:

23. Soergel E. 1961 Die Vogelreste. In: Ewald Schuldt (ed.) *Hohen Viecheln. Ein mittelsteinzeitlicher Wohnplatz in Mecklenburg*. Deutsche Akademie der Wissenschaften zu Berlin, Schriften der Sektion für Vor- und Frühgeschichte, Band 10. Berlin: Akademie-Verlag, pp. 64–69.

28. Driesch A. von den und Boessneck J. 1969 Die Fauna des Cabezo Redondo bei Villena (Prov. Alicante). In *Studien über frühe Tierknochenfunde von der Iberischen Halbinsel*, Band 1, S. 43–89 (oder 43–95 je nach Zitation). München: Institut für Palaeoanatomie, Domestikationsforschung und Geschichte der Tiermedizin der Universität München / Deutsches Archäologisches Institut (Abteilung Madrid).

106. King J. M. 1987 The animal bones. In *Longthorpe II: The Military Works-Depot: An Episode in Landscape History* (eds GB Dannell and JP Wild). London: Society for the Promotion of Roman Studies, Britannia Monograph Series No. 8.

107. Thawley C. R. 1981 The mammal, bird and fish bones. In *The Austin Friars, Leicester*. (eds JE Mellor and T Pearce). London: Council for British Archaeology,C BA Research Report 35.

Q17:

29. Hamilton-Dyer S. 1993 The animal bone. In Excavations in the Scamnum Tribunorum at Caerleon: The Legionary Museum Site 1983–5. (ed JD Zienkiewicz). *Britannia*, 24, 132–136

45. Pétillon J-M *et al.* 2023 Continuities and discontinuities in the human settlement of the Aquitaine Basin between 19.5 cal. ka BP and 13.5 cal. ka BP: The radiocarbon evidence. In *Hiatus, lacunes et absences : identifier et interpréter les vides archéologiques. Actes du 29e Congrès préhistorique de France, Toulouse, 31 mai-4 juin 2021*. Session *Les espaces vides: preuves d'absences ou absences de preuves?* (dirigée par Pétillon J-M, Cauliez J). [Société préhistorique française](https://www.google.co.uk/url?sa=i&source=web&rct=j&url=https://www.prehistoire.org&ved=2ahUKEwiElsb3zoGXAxV9UUEAHfKJHbkQy_kOegoIAggACAAIChAR&opi=89978449&cd&psig=AOvVaw1Wj7Xo8d0fBOCdovH9e3oo&ust=1790146878434000), Paris, pp. 47–64.

87. Müller M, Rudnick B, Zieling N, Ullrich . 2024 *Colonia Ulpia Traiana, Xanten: die geophysikalischen Untersuchungen mit GPR der Jahre 2006 bis 2023*. [Xantener Berichte, Band 47](https://www.google.co.uk/url?sa=i&source=web&rct=j&url=https://www.amazon.de/Xantener-Berichte-Band-geophysikalischen-Untersuchungen/dp/3961763054&ved=2ahUKEwjD3aiElP2WAxWxR_4FHR9rLsoQy_kOegoIAggACAAIChAH&opi=89978449&cd&psig=AOvVaw1TteE2-tfK9bzcT1TKp-BZ&ust=1789993628569000). Mainz / Nünnerich-Asmus Verlag & Media.

Q19:

76. Pétillon J-M. 2024 Sainte-Colome, grotte Tastet, fouille 2021. *Bilan scientifique de la région Nouvelle-Aquitaine – Année 2021*, Service régional de l'archéologie de la DRAC Nouvelle-Aquitaine, pp. 410-11.

77. Langlais M, Pétillon J-M. 2019 Les Pyrénées, une frontière pré-historiographique pour le Magdalénien? Réflexions àpartir du Magdalénien moyen récent de la grotte Tastet (Sainte-Colome, Pyrénées-Atlantiques). In *La conquête de la montagne: des premières occupations humaines à l’anthropisation du milieu*, *actes du 142ème congrès du CTHS, avril 2017, pau*. (eds M Deschamps, S Costamagno, P-Y Milcent, J-M, Pétillon, C Renard, N Valdeyron). Psaris: Éditions du Comité des travaux historiques et scientifiques

**Other issues:**

In the “3.1. Morphometric analysis” section:

“The two humeri sampled (#6247 –PE45 and #6250 –PE48) had measurements in the range of modern males or females (table 3)”. – Needs to be Table 3 and 4.

“The Xanten femur (PE32) had a single measurement (Bp) that fell in the overlap range of the modern male and female *G. grus* ( tables 4 and 4 )” - Needs to be Table 3 and 4.

In the “3.1.2. Sex estimation” section:

“Molecular sex estimation was possible for 22 of the fossil specimens. Twenty of these were confidently assigned as either male or female based on their Z:A coverage ratio, in accordance with the classification thresholds of Cabrera *et al.* [103 ] (supplementary material, tables 3 and S1).” – Table 3 should be table 4.

In Introduction:

“Extant male *G. grus* are significantly larger than females (44)” – That should be reference 55

“*A. antigone* var. primigenia” – the primigenia needs italics.

In Table 1:

For the Longthorpe site - [105 in [55 ] should be just [106 in 57]

For Austin Friars - [106 in [55] should be just [107 in 57]

In the “2.3. Extraction of ancient DNA, library preparation and whole-genome sequencing” section:

“The extraction was eluted in 50 μl TET (Tris-EDTA + 0.05% Tween). We then built double-stranded Illumina libraries according to Meyer and Kircher (88)” – the reference is [105].

In Table 3:

Do we need the Key and the Notes? They are the same information. – Suggest removing the Key as the Notes has the Reference.

Also in Table caption: quaternary should be Quaternary – with capital Q.

References:

18. Marjorie Northcote E, Mourer-ChauviréC. 1988 The extinct crane *Grus primigenia* Milne-Edwards in Majorca (Spain). *Geobios* **21** , 201–208.

Should be:

18. Northcote ME, Mourer-Chauviré C. 1988 The extinct crane *Grus primigenia* Milne-Edwards in Majorca (Spain). *Geobios* **21**, 201–208.

[First name moved and changed to initial]

27. Northcote EM. 1982a Sympatry of common cranes, *Grus grus* with larger Cranesin the last c. 125,000 years. *Bull. Brit. Orn. Club* **102** , 141–142.

Should be:

27. Northcote EM. 1982a Sympatry of common cranes, *Grus grus* with larger Cranes in the last c. 125,000 years. *Bull. Brit. Orn. Club* **102** , 141–142.

[Space added between ‘cranes’ and ‘in’]

44. Laroulandie V. 2023 Biographie d’un fémur de Grue. Bioarchéologies 04/12/2023.

44. Laroulandie V. 2023 Biographie d’un fémur de Grue. *Bioarchéologies* 04/12/2023.

[*Bioarchéologies* in italics]

63. Rabiniak E, Rekovets L, Stewart JR, Dalén L, Barton N, Strzala T, Barkaszi Z, Kovalchuk O. 2023 Late Pleistocene and Holocene pikas (Mammalia, Lagomorpha) from Europe and the validity of ochotona spelaea: new insights based on mtDNA analysis *. Palaeontol. Electron.* **26**, a3.

63. Rabiniak E, Rekovets L, Stewart JR, Dalén L, Barton N, Strzala T, Barkaszi Z, Kovalchuk O. 2023 Late Pleistocene and Holocene pikas (Mammalia, Lagomorpha) from Europe and the validity of *Ochotona spelaea*: new insights based on mtDNA analysis *. Palaeontol. Electron.* **26**, a3.

[*Ochotona spelaea* in italics]

81. Gourichon L. 1994 Les harfangs (Nyctea scandiaca L.) du gisement magdalénien du morin (Gironde). Analyse taphonomique des restes d’un rapace nocturne chasséet exploitépar les hommes préhistoriques. *Mémoire de DEA* : UniversitéLumière-Lyon 2, 180 p.

81. Gourichon L. 1994 Les harfangs (*Nyctea scandiaca* L.) du gisement magdalénien du morin (Gironde). Analyse taphonomique des restes d’un rapace nocturne chasséet exploitépar les hommes préhistoriques. *Mémoire de DEA* : UniversitéLumière-Lyon 2, 180 p.

[*Nyctea scandiaca* in italics]
